# MCM family in HCC: MCM6 indicates adverse tumor features and poor outcomes and promotes S/G2 cell cycle progression

**DOI:** 10.1186/s12885-018-4056-8

**Published:** 2018-02-20

**Authors:** Zhikun Liu, Jie Li, Jun Chen, Qiaonan Shan, Haojiang Dai, Haiyang Xie, Lin Zhou, Xiao Xu, Shusen Zheng

**Affiliations:** 1 Division of Hepatobiliary and Pancreatic Surgery, Department of Surgery, First Affiliated Hospital, Zhejiang University School of Medicine, Hangzhou, China; 2 Key Lab of Combined Multi-Organ Transplantation, Ministry of Public Health, Beijing, China; 3 Collaborative innovation center for diagnosis and treatment of infectious diseases, Hangzhou, China

**Keywords:** Hepatocarcinogenesis, HCC, MCMs, MCM6

## Abstract

**Background:**

Minichromosome Maintenance family (MCMs), as replication licensing factors, is involved in the pathogenesis of tumors. Here, we investigated the expression of MCMs and their values in hepatocellular carcinoma (HCC).

**Methods:**

MCMs were analyzed in 105 samples including normal livers (*n* = 15), cirrhotic livers (*n* = 40), HCC (*n* = 50) using quantitative polymerase chain reaction (qPCR) (Cohort 1). Significantly up-regulated MCMs were verified in 102 HCC and matched peritumoral livers using PCR (Cohort 2), and the correlations with clinical features and outcomes were determined. In addition, the focused MCMs were analyzed in parallel immunohistochemistry of 345 samples on spectrum of hepatocarcinogenesis (Cohort 3) and queried for the potential specific role in cell cycle.

**Results:**

MCM2–7, MCM8 and MCM10 was significantly up-regulated in HCC in Cohort 1. In Cohort 2, overexpression of MCM2–7, MCM8 and MCM10 was verified and significantly correlated with each other. Elevated MCM2, MCM6 and MCM7 were associated with adverse tumor features and poorer outcomes. In Cohort 3, MCM6 exhibited superior HCC diagnostic performance compared with MCM2 and MCM7 (AUC: 0.896 vs. 0.675 and 0.771, *P* < 0.01). Additionally, MCM6 other than MCM2 and MCM7 independently predicted poorer survival in 175 HCC patients. Furthermore, knockdown of MCM6 caused a delay in S/G2-phase progression as evidenced by down-regulation of CDK2, CDK4, CyclinA, CyclinB1, CyclinD1, and CyclinE in HCC cells.

**Conclusions:**

We analyze MCMs mRNA and protein levels in tissue samples during hepatocarcinogenesis. MCM6 is identified as a driver of S/G2 cell cycle progression and a potential diagnostic and prognostic marker in HCC.

**Electronic supplementary material:**

The online version of this article (10.1186/s12885-018-4056-8) contains supplementary material, which is available to authorized users.

## Background

Hepatocarcinogenesis is a typical stepwise process evolving from normal hepatocytes through chronic cirrhosis to hepatocellular carcinoma (HCC) [1, 2]. In China, there are 93 million hepatitis B virus (HBV) carriers which is 7.2% of the entire population, and HCC patients account for over 55% of new HCC cases worldwide [3, 4]. Sustained inflammation caused by HBV infection contributes to the majority etiology of HCC, and thus presents the opportunity to use liver samples from different stages of HBV-triggered disease to study the interesting genes or proteins suspected to be involved in hepatocarcinogenesis.

DNA replication is the scientific interest in studying the development and progression of tumor. The minichromosome maintenance family (MCMs) plays a central role in the replication, as replicative DNA helicase, and forms a hexameric ring-shaped complex around DNA. MCM proteins were first recognized in the yeast *Saccharomyces cerevisiae* and are essential for DNA replication in all eukaryotic cells, playing an important role in limiting replication in each cell cycle [5]. At least 10 homologues have been characterized in humans. Among these, the MCM2–7 complex participates in the pre-replication complex formation and exhibits helicase activity which makes DNA unwind, and resulting in recruitment of DNA polymerases and initiation of DNA replication and elongation [6, 7]. MCM8 is associated with chromosomal instability [8]. MCM10 mediates the interaction between RECQL4 and the MCM2–7 complex during DNA replication [9]. The twisted and tilted single hexamer shape of MCMs suggests a concerted mechanism that requires the structural deformation of the intervening DNA [10]. MCMs are essential for DNA replication in dividing cells and are lost in quiescence.

MCMs are candidate markers for cell proliferation, and increased levels of MCMs indicate proliferation of malignant cells. An increasing body of evidence suggests that MCMs predict tumor progression and prognosis. MCMs have been reported to be abnormally expressed in multiple malignancies including cervical cancer [11], breast cancer [12], and human gliomas [13]. Some MCMs have been found to be associated with disease prognosis. MCM2 is a useful marker in screening for cervical carcinoma [14], oral squamous cell carcinoma [15] and medulloblastoma [16], and it serves as a therapeutic target of the drug lovastatin in non-small cell lung carcinomas [17]. MCM3 may be a better marker than Ki-67 for the evaluation of dysplastic oral lesions [18]. A mutation of MCM4 is detected in skin cancer cells, which affects the DNA helicase activity of the MCM2–7 complex [19]. MCM5 is associated with breast cancer prognosis [20]. MCM7 contributes to the invasive capacity of papillary urothelial neoplasia [21] and is a risk factor for recurrence in patients with Dukes C colorectal cancer [22].

There are also isolated reports of the deregulated expression of individual MCMs in HCC. MCM2 is associated with the progression from cirrhosis to HCC and poor cellular differentiation [23, 24]. Serum MCM6 levels have been reported as the promising independent biomarker for HCC, especially in AFP negative and small HCC patients [25]. An immunohistochemical study has shown that MCM7 is increased in HCC [26]. Silencing of MCM7 with shRNA inhibits the malignant behavior of HCC cells via cell cycle arrest and apoptosis [27]. However, the other members of MCMs remain poorly understood in HCC. The comparison among MCMs in HCC has not yet been performed. Hepatocarcinogenesis is a typical multistage process characterized by chronic viral infection, cirrhosis, and HCC [28, 29]. Here, we feature the expression dynamics of MCM2, MCM3, MCM4, MCM5, MCM6, MCM7, MCM8, MCM9, MCM10 and RecQ like helicase 4 (RECQL4) in the typical multistep of hepatocarcinogenesis and demonstrate the association between MCMs and clinicopathological characteristics, diagnosis and prognosis in HCC patients.

## Methods

### Patients and samples

The samples used in this study were categorized into three independent Cohorts. Cohort 1 was used to profile the mRNA expression of MCMs and included 105 samples (15 normal livers, 40 HBV cirrhotic livers and 50 HBV-related HCC). Cohort 2 consisted of 102 HBV-related HCC and matched pritumoral livers, and it was used to investigate the clinical implications of MCMs. The samples of Cohort 1 and 2 were immediately snap-frozen in liquid nitrogen after surgical excision and stored at − 80 °C. The tissue microarrays HLiv-HCC1805ur-02 and HLiv-HCC1805ur-03 (OUTDO BIOTECH CO., LTD, China) and part of formalin-fixed paraffin-embedded samples in our hospital were used for immunohistochemistry as Cohort 3 (*n* = 345). Cohort 3 contained various lesion types (60 normal livers, 110 HBV cirrhotic livers, and 175 HCC). And the major etiology of HCC in cohort 3 were HBV but the accurate proportion was unclear. The normal hepatic samples were from the patients who underwent operation for hemangioma. A diagnosis of cirrhosis was defined histologically as having fibrosis stage 5/6 [30]. The cirrhotic livers were from HCC-absence cirrhotic patients who underwent liver transplantation. The diagnosis of HCC was made by pathological examination of the resected tissues. Approval for these studies was obtained from the Ethics Committee of the First Hospital of Zhejiang University, and all subjects in this study provided written informed consent. All aspects of the study related to human participants were in accordance with the ethical standards of the national research committee as well as with the Helsinki declaration.

### Quantitative reverse transcription polymerase chain reaction

Total RNA was isolated from tissue samples preserved at − 80 °C using Trizol (Invitrogen, USA). Good quality RNA (as confirmed by the integrity of 28S and 18S rRNA on agarose gel and A260/A280 ratio) was reverse transcribed by the cDNA kit (vazyme, China) according to manufacturer’s protocol. Quantitative polymerase chain reaction (qPCR) assays were performed using the ABI 7500 fast system (Applied Biosystems, USA). The gene specific primers are shown in Additional file 1. Gene expression was measured in triplicate in the optimized PCR condition as described previously [31]: one cycle of denaturing at 95 °C for 3 min, followed by 40 cycles of amplification at 95 °C for 15 s and 60 °C for 30 s, and last cycle along the melting curve at 95 °C for 15 s, 60 °C for 15 s and 95 °C for 15 s. Relative expression of genes was normalized to GAPDH and reported as 2-△CT, and △CT = Ct(target gene)-Ct(GAPDH).

### Immunocytochemistry

Four μm thick sections of samples were cut and mounted on poly L-lysine coated slides. Expression of the MCM2, MCM6 and MCM7 proteins were detected in paraffin-embedded samples in Cohort 3. As described previously [32], the sections were de-waxed and antigen retrieval was performed. After blanching of endogenous peroxidase, the sections were blocked and then incubated with the primary antibody at 4 °C overnight, and subsequently washed by PBS buffer at room temperature. On the next day, the slides were incubated with the secondary antibody (Biotech Inc., China) for 60 min at room temperature and the DAB detection was followed by Mayer’s haematoxylin nuclei counterstaining. Immunoreactivity score was assessed semi-quantitatively by determining the number of positive cells over the total number of liver cells: 0%, 5%, 10%, up to 100%, as reported [32, 33]. The assessment was performed by two independent pathologists in a double-blind manner. The antibodies and the dilution were detailed in the Additional file 2.

### Analysis of cell cycle distribution

Human HCC cell line Huh 7 (TCHu182) was purchased from the Institute of Biochemistry and Cell Biology, Chinese Academy of S ciences (Shanghai, China) and maintained in Dulbecco’s Modified Eagle’s Medium supplemented with 10% fetal bovine serum (Gibco, CA, USA) in a 37 °C incubator with 5% CO_2_. The cells in logarithmic growth phase were harvested, seeded into 6-well plates (2 × 10^5^/well) and transfected with Si-MCM6 or SiRNA control (Additional file 3). After 48 h, the cells were collected for flow cytometry. This experiment was repeated three times. For the detailed methods, please refer to the previous publication [34].

### Statistical analysis

Data were described as qualitative or quantitative variables. Qualitative data were compared with Fisher’s exact or Pearson’s chi-squared test, and quantitative ones with Student’s t test or Variance analysis, where appropriate. The correlations were analyzed by Kendall. Receiver operating characteristic curves (ROC) were performed to assess the diagnostic value of candidate proteins. Survival curve was plotted using Kaplan-Meier method and compared by the log-rank test. The independent factors of survival were identified using Cox’s proportional hazards model. Statistical analysis was performed with SPSS version 18.0 (SPSS, Chicago, IL, USA). Two-sided *P*-values of < 0.05 were considered to be significant.

## Results

### mRNA dynamics of MCMs in multistep hepatocarcinogenesis

**Fig. 1:**
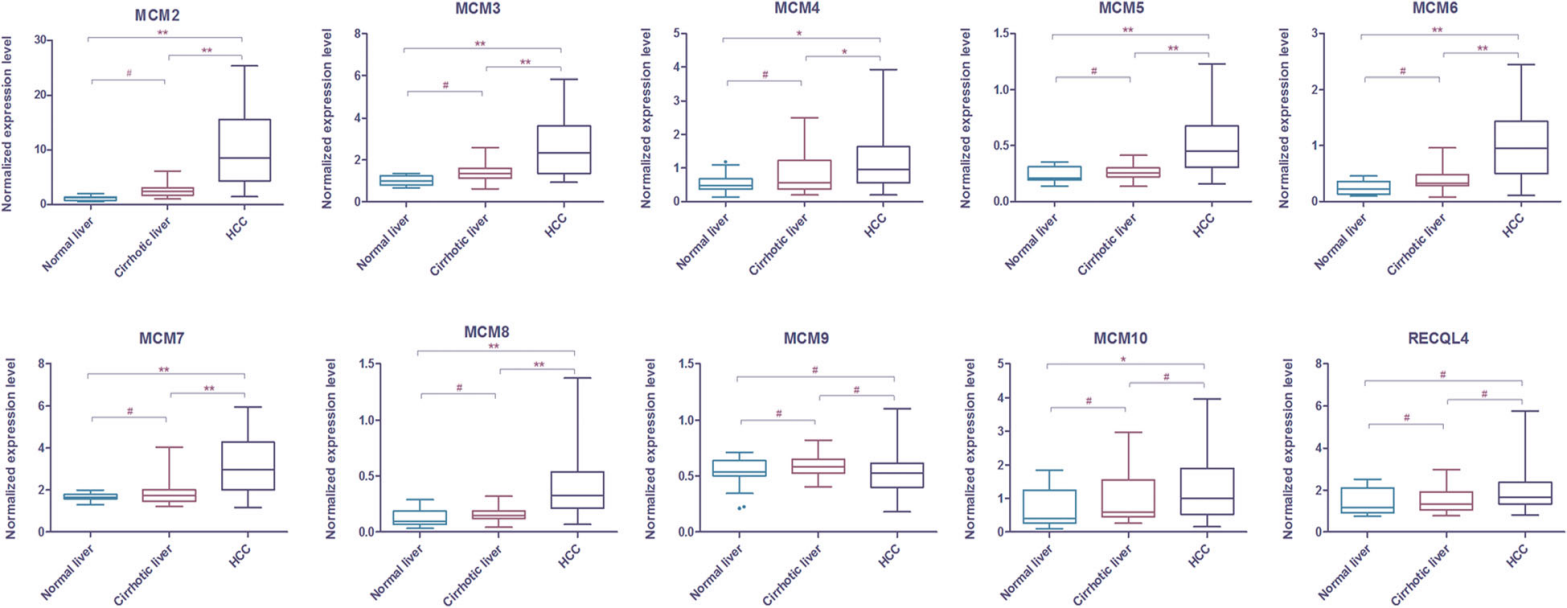
mRNA dynamics of MCM2–7, MCM8, MCM9, MCM10 and RECQL4 in multistep hepatocarcinogenesis. Box plots represent the distribution of normalized expression values of the indicated genes in normal liver (n = 15), cirrhotic liver (n = 40) and HCC (n = 50). A box in a given box plot represents the interquartile range (25th percentile to 75th percentile), the middle line denotes the median and the extreme ends of the whiskers marks the minimum and maximum values. *, *P* < 0.01, ** < 0.001, #, *P* > 0.05

The mRNA profiles of MCM2–7, MCM8, MCM9, MCM10 and RECQL4 were investigated in the Cohort 1 (normal livers, *n* = 15; cirrhotic livers, *n* = 40; and HCC, *n* = 50) using qPCR. As shown in Fig. 1, mRNA levels of MCM2–7, MCM8 were significantly up-regulated in HCC compared to normal or cirrhotic livers, and there was no significant difference in expression in normal versus cirrhotic livers. MCM10 was only significantly up-regulated in HCC relative to normal livers. However, MCM9 and RECQL4 remained unchanged throughout the process of hepatocarcinogenesis. The relative changes, medians and interquartile ranges (25th percentile to 75th percentile) of all MCMs in each sample type were reported in Additional file 4. The top three up-regulated MCMs were MCM2, MCM6 and MCM8, which were up-regulated 4.57-, 3.11- and 2.79-fold in HCC relative to noncancerous liver, respectively. These data indicate that MCM2–7, MCM8 and MCM10 mRNA levels increase in HCC, and are candidate drivers of hepatocarcinogenesis.

### mRNA expression of MCM2–7, MCM8 and MCM10 in HCC and their clinical implications

**Fig. 2:**
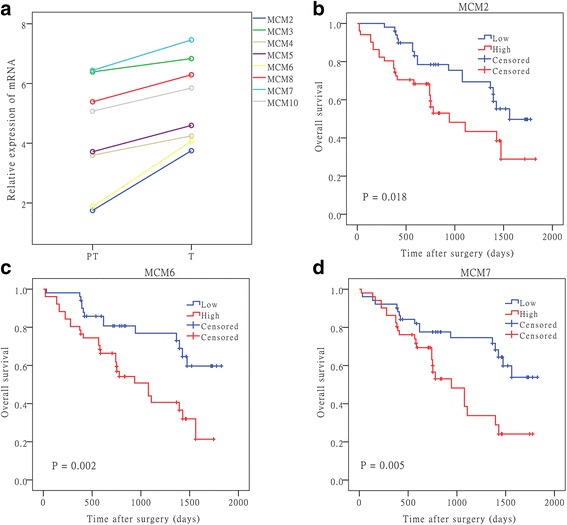
Elevated MCM2–7, MCM8, MCM10 and their prognostic implications in HCC. **a**, MCM2–7, MCM8 and MCM10 mRNA expression levels were analyzed in 102 HCC and matched peritumoral livers using RT-PCR and all molecules were significantly up-regulated (*P* < 0.01). N, peritumoral livers. MCM2 (**b**), MCM6 (**c**) and MCM7 (**d**) were associated with poor outcomes in HCC patients

The aberrant expression of MCM2–7, MCM8 and MCM10 in mRNA levels and their clinical relevance in HCC patients were further studied in HCC and matched peritumoral livers in the Cohort 2 (*n* = 102). We observed the similar results as in Cohort 1, mRNA levels of MCM2–7, MCM8 and MCM10 all significantly up-regulated in HCC than peritumoral livers (Fig. 2a). First, we investigated the correlations between the expression levels of MCMs. As shown in Additional file 4, the mRNA expression levels of these MCMs were significantly positively correlated with each other (Kendall correlation test, *P* < 0.05). These results indicate that the MCMs may be transcriptionally regulated together. Indeed, MCM members MCM2–7 are known to work as a complex to regulate DNA replication. Next, their clinical implications were also analyzed. As shown in Additional file 5, TNM stage correlated with MCM2–4, MCM6, MCM7 and MCM10, AFP was associated with MCM2, MCM4, MCM6 and MCM7. We proceeded to investigate whether mRNA levels of MCMs could predict the prognosis of HCC patients. Kaplan-Meier plots showed that patients with high MCM2, MCM6 and MCM7 expression had poorer outcomes (*P* = 0.018, 0.002, and 0.005, respectively; Fig. 2b-d). There was no correlation between other MCM mRNA levels and patient outcome (data not shown). These results suggest that MCM2, MCM6 and MCM7 mRNA levels could be potential prognostic markers for human HCC.

### Protein levels of MCM2, MCM6, and MCM7 as diagnostic and prognostic indicators for HCC

**Fig. 3:**
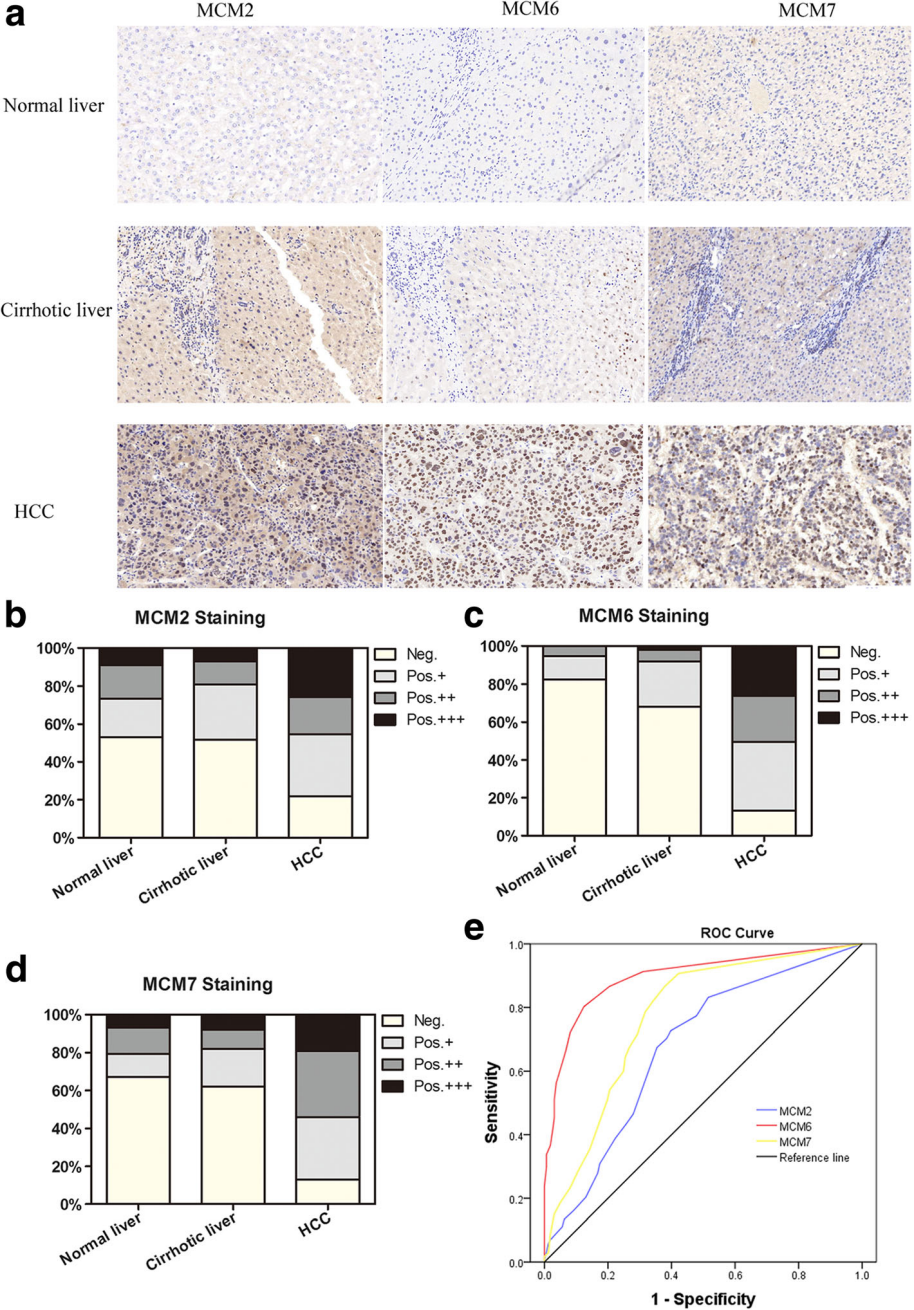
MCM2, MCM6 and MCM7 immunohistological testing in normal livers (*n* = 60), cirrhotic livers (*n* = 110), and HCC (*n* = 175). **a** Tissue MCM2, MCM6 and MCM7 immunoreactivity: from negative in normal livers to weak in cirrhotic livers to strong in HCC. The patterns of MCM2 (**b**), MCM6 (**c**) and MCM7 (**d**) expression in normal livers, cirrhotic livers and HCC were present according to the immunoreactive intensity. All three markers were all significantly higher in HCC (*P* < 0.01). **e** ROC curves comparing MCM2 (AUC = 0.675, *P* < 0.001), MCM6 (AUC = 0.896, *P* < 0.001), and MCM7 (AUC = 0.771, *P* < 0.001) in HCC versus normal and cirrhotic livers. ROC, receiver operating characteristics; Neg., negative; Pos., positive

To examine whether MCM2, MCM6 and MCM7 proteins are also exclusively overexpressed in HCC, we analyzed their expression patterns in Cohort 3 using immunohistochemistry. We detected them with antibodies in 60 normal livers, 110 cirrhotic livers and 175 HCC. The immunoreactivities of MCM2, MCM6 and MCM7 proteins were observed primarily in the hepatocellular cell nucleus and partly in the cytoplasm (Fig. 3a). Immunohistochemistry results were concordant with qPCR expression profiles. MCM2, MCM6 and MCM7 proteins were expressed at significantly higher levels in HCC compared to non-tumor specimens (*P* < 0.01; Fig. 3b-d), and the degree of their immunoreactivity gradually increased from normal and cirrhotic livers to HCC (Fig. 3b-d). These results suggest that increased MCM2, MCM6 and MCM7 proteins are associated with human HCC development. ROC curves were constructed to evaluate the area under the curve (AUC) for these potential diagnostic markers. The AUCs for MCM2, MCM6 and MCM7 proteins were 0.675, 0.896, and 0.771, respectively, and all the AUCs were significant compared with a Reference Line. MCM6 demonstrated optimal diagnostic performance, with an AUC significantly higher than that of MCM2 and MCM7 (Fig. 3e). These data indicate that MCM2, MCM6 and MCM7 proteins are potential diagnostic tissue markers for HCC, with MCM6 protein emerging as the primary candidate.

**Fig. 4:**
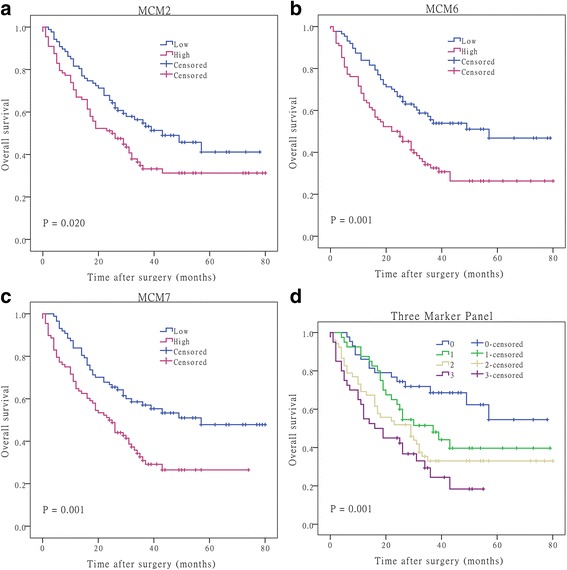
Kaplan-Meier survival curves with regard to overall survival according to MCM2, MCM6 and MCM7 protein expression in 175 patients with HCC (log-rank test). Specimens were stratified into high or low expression using the median expression level as the cut-off point. Overall survival of patients with high expression of MCM2 (**a**), MCM6 (**b**) and MCM7 (**c**) significantly worse than that with low expression (*P* = 0.020, 0.001 and 0.001, respectively). **d** Kaplan-Meier survival curve for specimens stratified into four groups based on the number of three-marker panel (MCM2, MCM6 and MCM7) expressed at a high level (*P* = 0.001). 0, 1, 2, and 3 indicates the number of MCMs expressed at a high level

Next, we determined the association between MCM2, MCM6, and MCM7 protein levels and specific pathologic features and outcomes in 175 HCC patients. Correlation analysis showed that MCM6 protein levels were significantly associated with Ki67 expression and differentiation, MCM7 with tumor size and Ki67 (*P* < 0.05, Table 1). However, no significant association between MCM2 protein levels and tumor characteristics such as tumor stage, tumor size, etc. was observed. Kaplan-Meier analysis showed that patients with high MCM2, MCM6 and MCM7 protein levels had significantly poorer prognosis than those with low expression (*P* = 0.020, 0.001, and 0.001, respectively; Fig. 4a-c). In addition, overexpression of multiple MCMs was found to be a very strong prognostic indictor (*P* = 0.001; Fig. 4d), as demonstrated when MCM2, MCM6 and MCM7 were combined (three-marker panel). These results strongly suggest that the combined use of MCM2, MCM6 and MCM7 is a reliable prognostic indicator for HCC patients. Finally, further univariate and multivariate Cox regression analyses revealed that MCM6 protein is a significant and independent predictor for poor outcome in HCC patients (Table 2).

**Table 1:** Relationship between MCM2, MCM6 and MCM7 expression and clinico-pathological characteristics in 175 HCC patients

	MCM2^a^			MCM6^a^			MCM7^a^		
Variables	Low	High	*p*	Low	High	*p*	Low	High	*p*
Age (years)			0.752			0.874			0.527
≤50	31	29		29	31		32	28	
> 50	56	59		58	57		55	60	
Sex			0.288			0.676			0.136
Female	10	16		14	12		9	17	
Male	77	72		73	76		78	71	
Tumor size (total diameter)			0.646			0.443			*0.021*
≤5 cm	37	34		38	33		43	28	
> 5 cm	50	54		49	55		44	60	
Tumor multiplicity			0.388			0.832			0.832
Single	77	73		74	76		74	76	
Multiple	10	15		13	12		13	12	
Tissue AFP^†^			0.880			0.228			0.451
Low	44	43		39	48		46	41	
High	43	45		48	40		41	47	
Tissue Ki67^†^			0.050			*< 0.001*			*0.008*
Low	50	37		62	25		52	35	
High	37	51		25	63		35	53	
Differentiation			0.057			*< 0.001*			0.259
Well	11	10		17	4		13	8	
Moderate	55	42		55	42		50	47	
Poor	21	36		15	42		24	33	
TNM stage			0.742			0.324			0.103
I	11	7		8	10		14	4	
II	37	37		43	31		38	36	
III	32	37		32	37		31	38	
IV	2	2		1	3		2	2	

^a^High and low expression were divided by the median expression level

Italic values indicate statistical significance

**Table 2:** Cox univariate and multivariate regression analyses of prognostic factors and MCM2, MCM6 and MCM7 expression for overall survival in HCC patients

Variables	Univariate Analysis		Multivariate Analysis	
	Hazard Ratio (95% CI)	*p* Value	Hazard Ratio (95% CI)	*p* Value
Age (> 50ys)	0.89(0.59–1.35)	0.590		
Gender (male)	1.24 (0.69–2.23)	0.464		
Total tumor size (> 5 cm)	2.26(1.47–3.49)	*< 0.001*	1.29(0.73–2.27)	0.390
Tumor multiplicity (multiple)	1.88 (1.14–3.10)	*0.014*	1.74(0.99–3.08)	0.056
Differentiation (mod./ well vs poor)	0.60 (0.40–0.89)	*0.012*	1.01(0.62–1.64)	0.974
TNM stage (I-II vs III-IV)	0.37 (0.24–0.57)	*< 0.001*	0.51(0.29–0.88)	*0.016*
Tissue AFP (High)	1.42(0.96–2.11)	0.081		
Tissue Ki67 (High)	1.74(1.16–2.59)	*0.007*	1.20(0.74–1.94)	0.471
MCM2 (High)	1.59(1.07–2.36)	*0.020*	1.19(0.74–1.91)	0.485
MCM6 (High)	1.93(1.29–2.88)	*0.001*	1.65(1.00–2.72)	*0.048*
MCM7 (High)	1.97(1.32–1.95)	*0.001*	1.50(0.94–2.39)	0.087

Italic values indicate statistical significance

### Cell cycle effect of MCM6 by flow cytometry

**Fig. 5:**
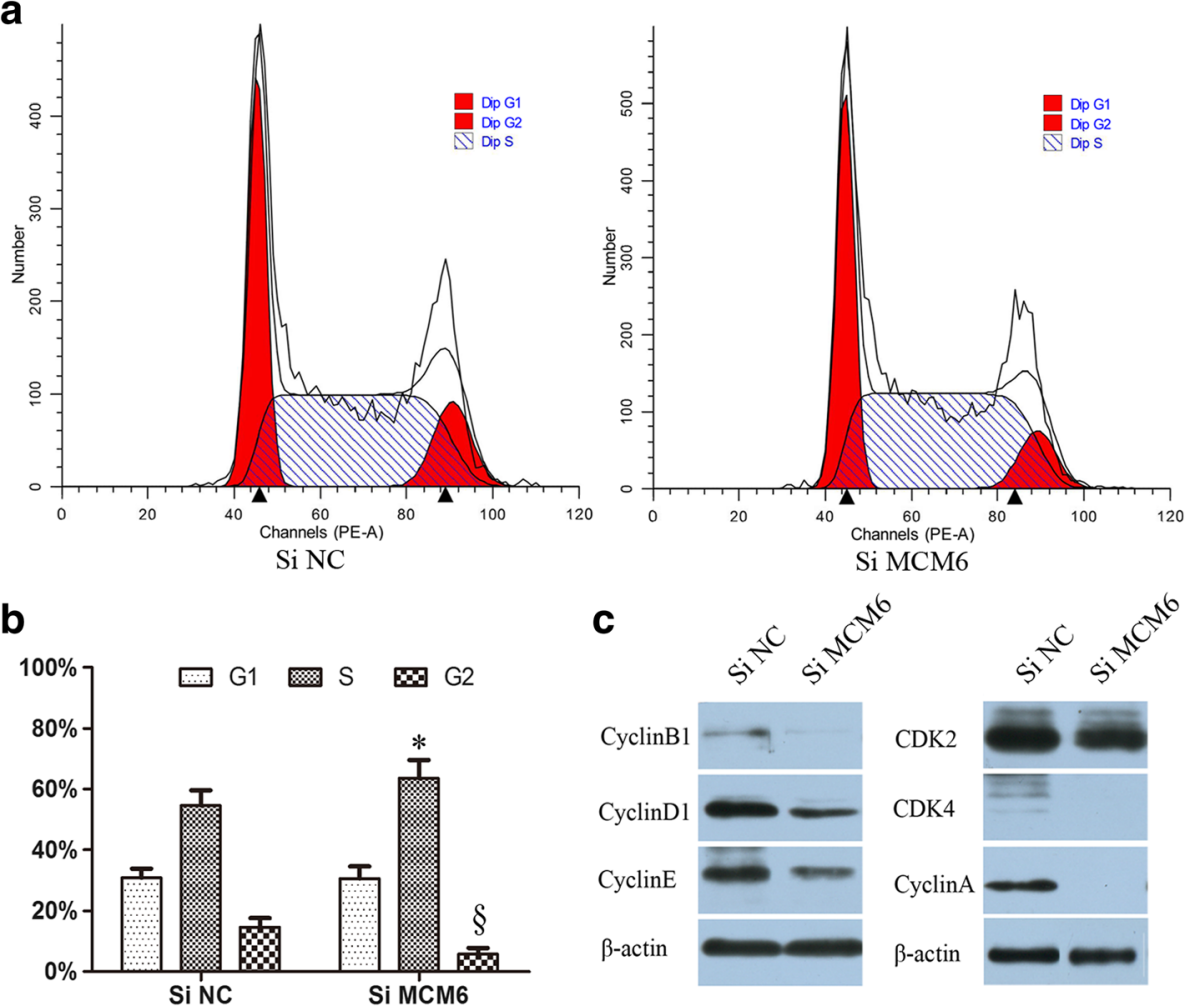
Cell cycle distribution affcted by MCM6. Huh7 cells tranfected with 50 nM Si-MCM6 or negative control (Si-NC) for 48 h. **a** The cell cycle distribution was determined by flow cytometry in Huh7. **b** Histograms obtained from flow cytometry measurements show arrest in the S phase in cells treated with Si-MCM6. **c** CDK2, CDK4, CyclinA, CyclinB1, CyclinD1, and CyclinE were lower in Si-MCM6 treated cells than Si-NC. Data were expressed as the mean of 3 independent experiments. * and§, *P* < 0.05 compared to control

Because MCMs play a central role in the replication of DNA, we further studied whether the potential effects of MCM6 on the cell cycle in Huh 7 cells. Compared with control group (54.6 ± 5.1), the proportion of cells in S phase increased markedly (63.6 ± 6.0) while in G2 phase reduced dramatically in Si-MCM6 group (Fig. 5a and b). This suggests that cells in the Si-MCM6 group were arrested in the S phase and failed to enter the G2 phase. Mechanistically, CDK2, CDK4, CyclinA, CyclinB1, CyclinD1, and CyclinE were lower in Si-MCM6 treated cells (Fig. 5c), suggesting that inhibition of MCM6 can delay the cell cycle S/G2 progression through down-regulating the cell cycle checkpoint.

## Discussion

Overexpression of MCMs is observed in many tumors, including cervical carcinoma [11], gliomas [13], oral squamous cell carcinoma [15, 35], and non-small cell lung carcinoma [36, 37]. In this study, we investigated the expression of MCMs in HCC via profiling their mRNA and protein levels. We further evaluated the MCMs with significantly up-regulated in HCC. Most of MCMs were showed to be up-regulated in HCC, indicating their key role in tumor cells. MCM6 were finally identified as novel candidate markers for HCC.

HCC usually occurs in the background of hepatic cirrhosis as a result of chronic hepatitis in Asia. We first presented an overview of the expression patterns of all MCMs on the spectrum of hepatocarcinogenesis. Within the MCM family, MCM2–7, MCM8 and MCM10 mRNAs were up-regulated during hepatocarcinogenesis. The increased expression of these mRNA was confirmed in Cohort 2. Interestingly, expression of certain MCMs was positively correlated with each other. It can be partial explained that MCM 2–7 form part of the pre-replicative complex to promote DNA synthesis [38]. Thus, MCM proteins function to allow the DNA replication machinery to access binding sites on DNA [39]. Despite the finding that many genes in the MCM family are up-regulated in HCC, only some of them in our study exhibited a critical role in hepatocarcinogenesis. Our results showed that mRNA levels of MCM2, MCM6 and MCM7 were associated with certain features of tumor and the outcomes of HCC patients. Meanwhile, the overexpression of MCM4, MCM5, MCM6, MCM10 and RECQL4 mRNA has been reported as a poor prognostic indicator in cervical cancer [11]. MCM2 was demonstrated as a biomarker for esophageal [40] and bladder cancer [41]. Furthermore, the increased levels of MCMs are not only useful for the initial diagnosis but can also predict tumor recurrence.

We further demonstrated increased protein levels of MCM2, MCM6 and MCM7 proteins in Cohort 3 by immunohistochemistry. Little was previously known about MCM2 and MCM6 protein expression in liver cancer, we observed their up-regulation in HCC. Of three markers, MCM6 protein showed the best diagnostic performance for HCC. Importantly, increased MCM2, MCM6 and MCM7 protein levels were associated with poorer survival in HCC patients. Consistent with the report by Zhou Y et al., MCM7 protein was associated with post-operative prognosis for HCC [26]. In our study, MCM6 protein showed the best diagnostic and prognostic marker of MCMs in HCC. Recent evidence confirms even higher levels of MCM6 in plasma as a novel biomarker for HCC patients [25]. We revealed strong positive correlations in the expression of MCM6 vs. Ki67, and MCM7 vs. Ki67 in HCC samples. Thus, MCM6 could reflect high rate of proliferation in HCC cells and may serve as a potential proliferation-specific marker for HCC. Our findings not only confirm the role of MCM7 in HCC, but also identify MCM2 and MCM6 as potential tissue diagnostic and prognostic markers for HCC. However, there are some shortcomings in our study: only including HBV-related HCC which is a major etiology of HCC in China but not the only cause of HCC; using one cell lines for cell cycle effect; lacking AFP data to be analyzed and compared with MCM6. The relevance of HBV virus infection to the MCM expression in HCC with the other etiological factors such as HCV or fatty liver needs to be worthy of further investigation.

## Conclusions

In summary, this study provides a comprehensive report of the expression profile of all MCMs in multistep hepatocarcinogenesis. The results offer an insight into the potential utility of these genes as proliferation-specific, diagnostic and prognostic markers for HCC. Further studies regarding the mechanism of MCMs in HCC may provide clues as to whether they can serve as potential therapeutic targets. Taken together, the present study has demonstrated the importance of MCM expression in HCC and that MCM6 could be a novel candidate prognostic and predictive indicator for HCC patients.

## Additional files

Additional file 1: Primer sequences. (DOC 38 kb)

Additional file 2: Antibodies and dilution used. (DOC 27 kb)

Additional file 3: SiNC and siMCM6. (DOC 27 kb)

Additional file 4: MCM mRNA expression patterns on the spectrum of hepatocarcinogenesis. (DOC 40 kb)

Additional file 5: Correlation between the expression levels of MCMs and their clinical implications in 102 patients with HCC. (DOC 47 kb)

Additional file 6: PCR data of cohort1. (XLS 59 kb)

Additional file 7 PCR data of cohort2. (XLS 46 kb)

## Abbreviations

AFP: alpha fetoprotein
AUC: areas under the receiver-operating characteristic curves
HBV: hepatitis B virus
HCC: hepatocellular carcinoma
MCMs: minichromosome maintenance family
Neg.: negative; pos., positive
PVTT: portal vein tumor thrombus
ROC: receiver operating characteristics
Tno.: number of tumors
