# Supplementary material for: MCM family in HCC: MCM6 indicates adverse tumor features and poor outcomes and promotes S/G2 cell cycle progression
Source: BMC Cancer. 2018 Feb 20;18:200. doi: 10.1186/s12885-018-4056-8 (PMC5819696; doi:10.1186/s12885-018-4056-8)
Supplement: Supplementary file 1 — Primer sequences. (DOC 38 kb) [file 12885_2018_4056_MOESM1_ESM.doc]

Additional file 1. Primer sequences

| Gene | Sequence (5’–3’) |
| --- | --- |
| GAPDH Forward | GTCTCCTCTGACTTCAACAGCG |
| GAPDH Reverse | ACCACCCTGTTGCTGTAGCCAA |
| MCM2 Forward | TGCCAGCATTGCTCCTTCCATC |
| MCM2 Reverse | AAACTGCGACTTCGCTGTGCCA |
| MCM3 Forward | CGAGACCTAGAAAATGGCAGCC |
| MCM3 Reverse | GCAGTGCAAAGCACATACCGCA |
| MCM4 Forward | CTTGCTTCAGCCTTGGCTCCAA |
| MCM4 Reverse | GTCGCCACACAGCAAGATGTTG |
| MCM5 Forward | GACTTACTCGCCGAGGAGACAT |
| MCM5 Reverse | TGCTGCCTTTCCCAGACGTGTA |
| MCM6 Forward | GACAACAGGAGAAGGGACCTCT |
| MCM6 Reverse | GGACGCTTTACCACTGGTGTAG |
| MCM7 Forward | GCCAAGTCTCAGCTCCTGTCAT |
| MCM7 Reverse | CCTCTAAGGTCAGTTCTCCACTC |
| MCM8 Forward | CTGTGTGTCGAGGCAGGTCATT |
| MCM8 Reverse | TCGTGGAATCCGACCTGCTTCT |
| MCM9 Forward | GGCTATCTGTTGGAAGTATTCCAC |
| MCM9 Reverse | TGCTGAAAGGGCTTCCACCGTT |
| MCM10 Forward | TCAAGGAACTGATGGACCTGCC |
| MCM10 Reverse | CTCCAACATCCGCTGCTTCTGT |
| RECQL4 Forward | AAGCAACGGGAATCTGTCCTGC |
| RECQL4 Reverse | CAGGCAAAAGCAACTGGAGGCA |
