# Supplementary material for: MCM family in HCC: MCM6 indicates adverse tumor features and poor outcomes and promotes S/G2 cell cycle progression
Source: BMC Cancer. 2018 Feb 20;18:200. doi: 10.1186/s12885-018-4056-8 (PMC5819696; doi:10.1186/s12885-018-4056-8)
Supplement: Supplementary file 2 — Antibodies and dilution used. (DOC 27 kb) [file 12885_2018_4056_MOESM2_ESM.doc]

Additional file 2: Antibodies and dilution

| Antibodies | Company | Dilution |
| --- | --- | --- |
| MCM2 | Proteintech | 1:200 |
| MCM6 | Epitomics | 1:150 |
| MCM7 | Proteintech | 1:200 |
