# Supplementary material for: MCM family in HCC: MCM6 indicates adverse tumor features and poor outcomes and promotes S/G2 cell cycle progression
Source: BMC Cancer. 2018 Feb 20;18:200. doi: 10.1186/s12885-018-4056-8 (PMC5819696; doi:10.1186/s12885-018-4056-8)
Supplement: Supplementary file 3 — SiNC and siMCM6. (DOC 27 kb) [file 12885_2018_4056_MOESM3_ESM.doc]

Additional file 3: SiNC and siMCM6

|  | Sequence (5’–3’) | Company |
| --- | --- | --- |
| SiNC | UUCUCCGAACGUGUCACGUtt | Novars |
| SiMCM6-01 | GUACUUGAAGGAAAUCGAAtt | Novars |
| SiMCM6-02 | CCCAAUCAGUGGACACUAUtt | Novars |
| SiMCM6-03 | GAAUUGAGGAAUCAAUUGAtt | Novars |
