# Supplementary material for: MCM family in HCC: MCM6 indicates adverse tumor features and poor outcomes and promotes S/G2 cell cycle progression
Source: BMC Cancer. 2018 Feb 20;18:200. doi: 10.1186/s12885-018-4056-8 (PMC5819696; doi:10.1186/s12885-018-4056-8)
Supplement: Supplementary file 4 — MCM mRNA expression patterns on the spectrum of hepatocarcinogenesis. (DOC 40 kb) [file 12885_2018_4056_MOESM4_ESM.doc]

Additional file 4. MCM mRNA expression patterns on the spectrum of hepatocarcinogenesis

| MCM family | Normal livers | | Cirrhotic livers | | HCC | | Fold change  HCC/normal+cirrhotic livers |
| --- | --- | --- | --- | --- | --- | --- | --- |
|  | median interquartile range | mean±SD | median interquartile range | mean±SD | median interquartile range | mean±SD |  |
| MCM2 | 1.24 (0.80-1.40) | 1.21±0.43 | 2.41 (1.74-3.10) | 2.56±1.08 | 8.49 (5.34-15.63) | 10.0±6.72 | 4.57 |
| MCM3 | 0.98 (0.80-1.24) | 1.02±0.23 | 1.34 (1.13-1.61) | 1.41±0.39 | 2.32 (1.36-3.62) | 2.69±1.41 | 2.07 |
| MCM4 | 1.47 (0.36-0.68) | 0.54±0.29 | 0.56 (0.36-1.23) | 0.80±0.60 | 0.95 (0.55-1.63) | 1.19±0.78 | 1.63 |
| MCM5 | 0.21 (0.19-0.31) | 0.24±0.06 | 0.26 (0.22-0.30) | 0.26±0.06 | 0.45 (0.31-0.67) | 0.53±0.27 | 2.06 |
| MCM6 | 0.22 (0.13-0.36) | 0.24±0.12 | 0.33 (0.28-0.48) | 0.38±0.16 | 0.95 (0.50-1.44) | 1.06±0.60 | 3.11 |
| MCM7 | 1.66 (1.57-1.78) | 1.66±0.20 | 1.73 (1.45-2.03) | 1.81±0.52 | 2.97 (2.00-4.28) | 3.24±1.33 | 1.83 |
| MCM8 | 0.09 (0.07-0.19) | 0.13±0.07 | 0.15 (0.12-0.19) | 0.16±0.06 | 0.33 (0.21-0.54) | 0.43±0.30 | 2.79 |
| MCM9 | 0.54 (0.50-0.64) | 0.52±0.15 | 0.58 (0.53-0.65) | 0.59±0.11 | 0.53 (0.40-0.62) | 0.53±0.19 | 0.93 |
| MCM10 | 0.40 (0.27-1.25) | 0.68±0.64 | 0.59 (0.46-1.55) | 0.94±073 | 1.01 (0.52-1.90) | 1.32±1.01 | 1.52 |
| RECQL4 | 1.17 (0.91-2.11) | 1.44±0.63 | 1.33 (1.05-1.92) | 1.50±0.57 | 1.66 (1.33-2.38) | 1.92±0.93 | 1.29 |

Interquartile range, 25th percentile to 75th percentile.
