# Supplementary material for: MCM family in HCC: MCM6 indicates adverse tumor features and poor outcomes and promotes S/G2 cell cycle progression
Source: BMC Cancer. 2018 Feb 20;18:200. doi: 10.1186/s12885-018-4056-8 (PMC5819696; doi:10.1186/s12885-018-4056-8)
Supplement: Supplementary file 5 — Correlation between the expression levels of MCMs and their clinical implications in 102 patients with HCC. (DOC 47 kb) [file 12885_2018_4056_MOESM5_ESM.doc]

Additional file 5. Correlation between the mRNA levels of MCMs and their clinical implications in 102 patients with HCC

| Correlation Coefficient | MCM2 | MCM3 | MCM4 | MCM5 | MCM6 | MCM7 | MCM8 | MCM10 | TNM | AFP | Tno. | Grade | PVTT |
| --- | --- | --- | --- | --- | --- | --- | --- | --- | --- | --- | --- | --- | --- |
| MCM2 | 1.000 |  |  |  |  |  |  |  |  |  |  |  |  |
| MCM3 | 0.368^**^ | 1.000 |  |  |  |  |  |  |  |  |  |  |  |
| MCM4 | 0.342^**^ | 0.413^**^ | 1.000 |  |  |  |  |  |  |  |  |  |  |
| MCM5 | 0.290^**^ | 0.474^**^ | 0.373^**^ | 1.000 |  |  |  |  |  |  |  |  |  |
| MCM6 | 0.767^**^ | 0.290^**^ | 0.291^**^ | 0.306^**^ | 1.000 |  |  |  |  |  |  |  |  |
| MCM7 | 0.730^**^ | 0.379^**^ | 0.398^**^ | 0.325^**^ | 0.814^**^ | 1.000 |  |  |  |  |  |  |  |
| MCM8 | 0.407^**^ | 0.315^**^ | 0.321^**^ | 0.392^**^ | 0.398^**^ | 0.359^**^ | 1.000 |  |  |  |  |  |  |
| MCM10 | 0.430^**^ | 0.566^**^ | 0.487^**^ | 0.498^**^ | 0.367^**^ | 0.466^**^ | 0.459^**^ | 1.000 |  |  |  |  |  |
| TNM | 0.363^**^ | 0.172^*^ | 0.171^*^ | 0.119 | 0.345^**^ | 0.347^**^ | 0.058 | 0.175^*^ | 1.000 |  |  |  |  |
| AFP | 0.313^**^ | 0.159 | 0.184^*^ | 0.014 | 0.313^**^ | 0.367^**^ | 0.026 | 0.147 | 0.304^**^ | 1.000 |  |  |  |
| Tno. | 0.008 | 0.081 | 0.053 | 0.124 | 0.113 | 0.118 | 0.081 | 0.040 | 0.127 | 0.219^*^ | 1.000 |  |  |
| Grade | -0.125 | -0.078 | 0.099 | -0.016 | -0.098 | -0.083 | 0.018 | -0.037 | -0.277^**^ | -0.156 | -0.102 | 1.000 |  |
| PVTT | 0.182^*^ | 0.181^*^ | 0.128 | 0.073 | 0.126 | 0.167^*^ | -0.095 | 0.009 | 0.437^**^ | 0.293^**^ | 0.138 | -0.291^**^ | 1.000 |

TNM staging, American Joint Committee on Cancer staging; AFP, alpha fetoprotein; Tno., number of tumors; PVTT, portal vein tumor thrombus; *, *P* < 0.05; **, *P* < 0.001. Kendall correlation analysis was performed.
